# Inverted Y incision and trans-sacral approach in retroperitoneal aggressive angiomyxoma: a case report

**DOI:** 10.1186/1752-1947-7-153

**Published:** 2013-06-10

**Authors:** Dae Gy Hong, Gun Oh Chong, Young Lae Cho, Il Soo Park, Ji Young Park, Yoon Soon Lee

**Affiliations:** 1 Gynecologic Cancer Center, Kyungpook National University Medical Center, 807 Hoguk-ro, Buk-gu, Daegu, 702-210, Republic of Korea; 2 Department of Pathology, Kyungpook National University Medical Center, 807 Hoguk-ro, Buk-gu, Daegu, 702-210, Republic of Korea

**Keywords:** Aggressive angiomyxoma, Surgery, Inverted Y incision

## Abstract

**Introduction:**

Aggressive angiomyxoma is a rare myxedematous mesenchymal tumor that mainly occurs in the female pelvis and perineum. The principle of treatment for aggressive angiomyxoma is surgical excision. The tumor can be removed by local excision alone when it occurs locally on the perineum. However, it cannot be completely excised by a perineal approach alone when it passes through the perineum and pelvic bone to extend into the retroperitoneal space.

**Case presentation:**

A 34-year-old Asian woman presented with a rapidly growing left perineal mass and swelling in the left gluteal region. The swelling was associated with a mild, dull pain in the left gluteal region. In the present case of bulky aggressive angiomyxoma extending to the perineum and retroperitoneal space, the authors made an inverted Y incision through the buttock, removed the coccyx and lower portion of the sacrum, and excised the retroperitoneal mass and perineal lesion through a perineal approach.

**Conclusion:**

The inverted Y incision and trans-sacral approach can provide easy access to deep retroperitoneal aggressive angiomyxoma and reduce damage to neighboring organs.

## Introduction

Aggressive angiomyxoma (AAA) is a rare myxedematous mesenchymal tumor that mainly occurs in the female pelvis and perineum. AAA was first described by Steeper and Rosai in 1983 [1]. The principle treatment for AAA is surgical excision. It is not easy to determine the tumor-free margin because of infiltration; thus, complete resection is difficult. The tumor can be removed by local excision alone when it occurs locally on the perineum. However, it cannot be completely excised by a perineal approach alone when it passes through the perineum and pelvic bone to extend into the retroperitoneal space [2,3].

Given that AAA usually manifests in the second to fourth decades of life and frequently extends into the retroperitoneal space, appropriate surgical methods should be selected to ensure a cosmetic incision and effective mass removal. In the present case, involving a bulky AAA extending to the perineum and retroperitoneal space, the authors made an inverted Y incision through the buttock, removed the coccyx and lower portion of the sacrum, and excised the retroperitoneal mass and perineal lesion through a perineal approach without laparotomy.

## Case presentation

A 34-year-old Asian woman presented with a six-month history of a rapidly growing left perineal mass and swelling in the left gluteal region. The swelling was associated with a mild, dull pain in the left gluteal region.

**Figure 1:**
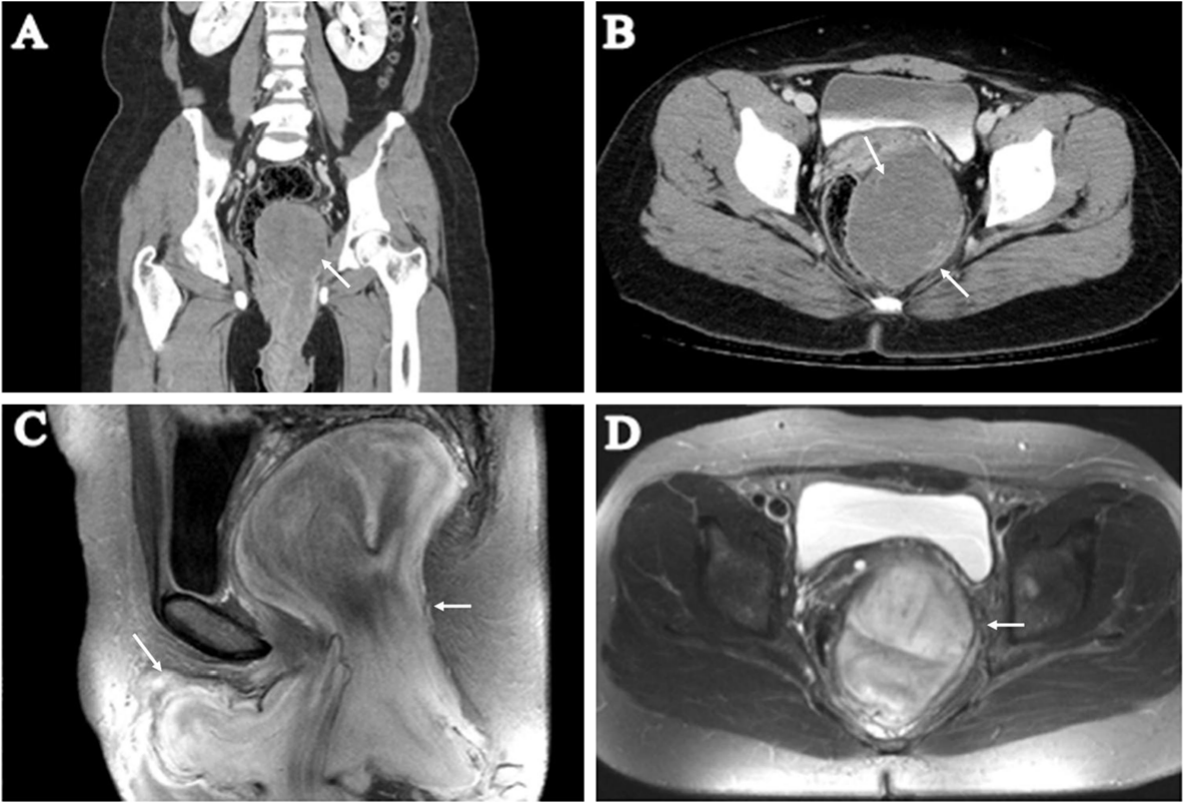
**Computed tomography and magnetic resonance imaging findings.** Computed tomography (**A**, coronal view; **B**, transverse view) shows a large tumor starting at the level of the third sacrum (S3) and pushing the distal colon toward the right; its distal extension ends at the perineal mass. Magnetic resonance imaging (**C**, T1-weighted image, sagittal view; **D**, T2-weighted image, transverse view) shows isointensity relative to muscle on T1-weighted imaging and hyperintensity relative to muscle on T2-weighted imaging. The white arrows in each image indicate the tumor and its margin.

Computed tomography (CT) showed a 40 × 10 × 12cm mass starting at the level of the third sacrum (S3) and pushing the distal colon toward the right; its distal extension ended at the perineal mass (Figure 1A, B). Magnetic resonance imaging (MRI) showed the same tumor seen on CT. The tumor was isointense relative to muscle on T1-weighted imaging (Figure 1C) and hyperintense to muscle on T2-weighted imaging (Figure 1D). The mass was very close to the sigmoid colon, rectum, and bladder. Because the mass was very large and bulky within the retroperitoneal space, preoperative size reduction was performed with a gonadotropin-releasing hormone (GnRH) analog. The GnRH analog was injected three times at one-month intervals, and a follow-up MRI was rechecked thereafter. There was no change in the size of the perineal mass, but its consistency had become slightly softer.

**Figure 2:**
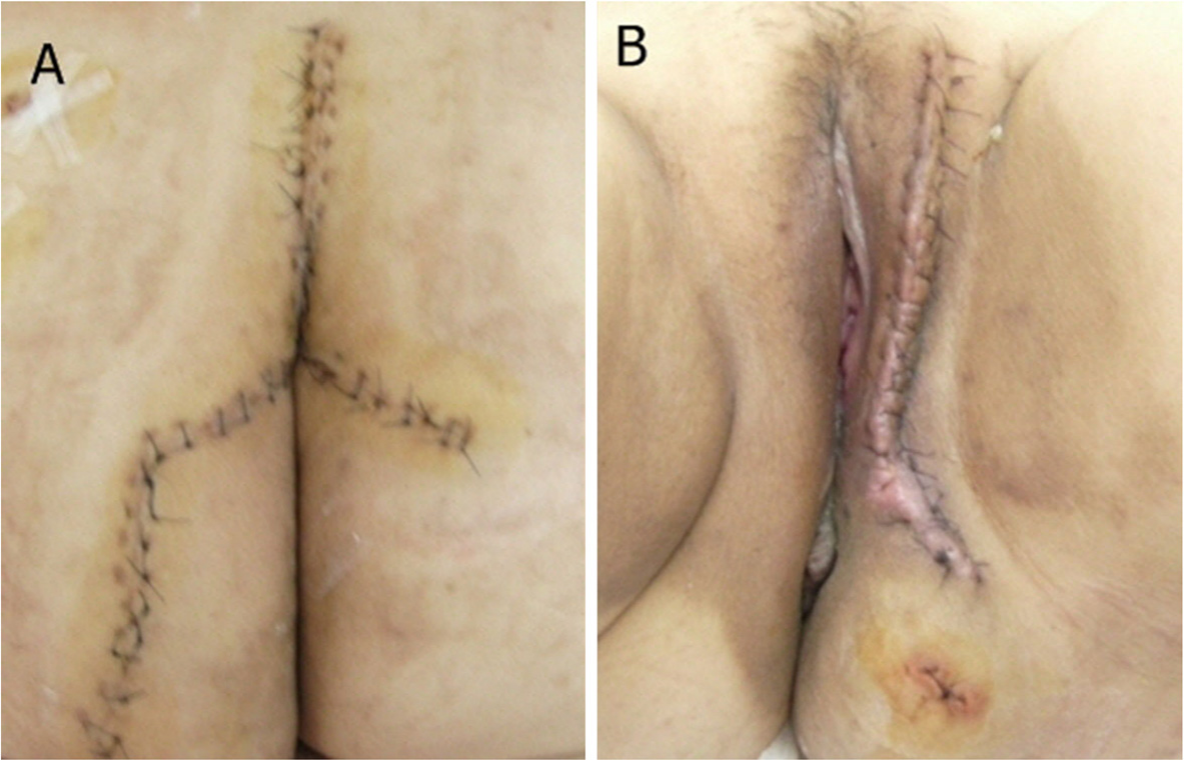
**Surgical incisions.** (**A**) Inverted Y incision centered on the lower portion of the sacrum. (**B**) Perineal incision on the left side.

**Figure 3:**
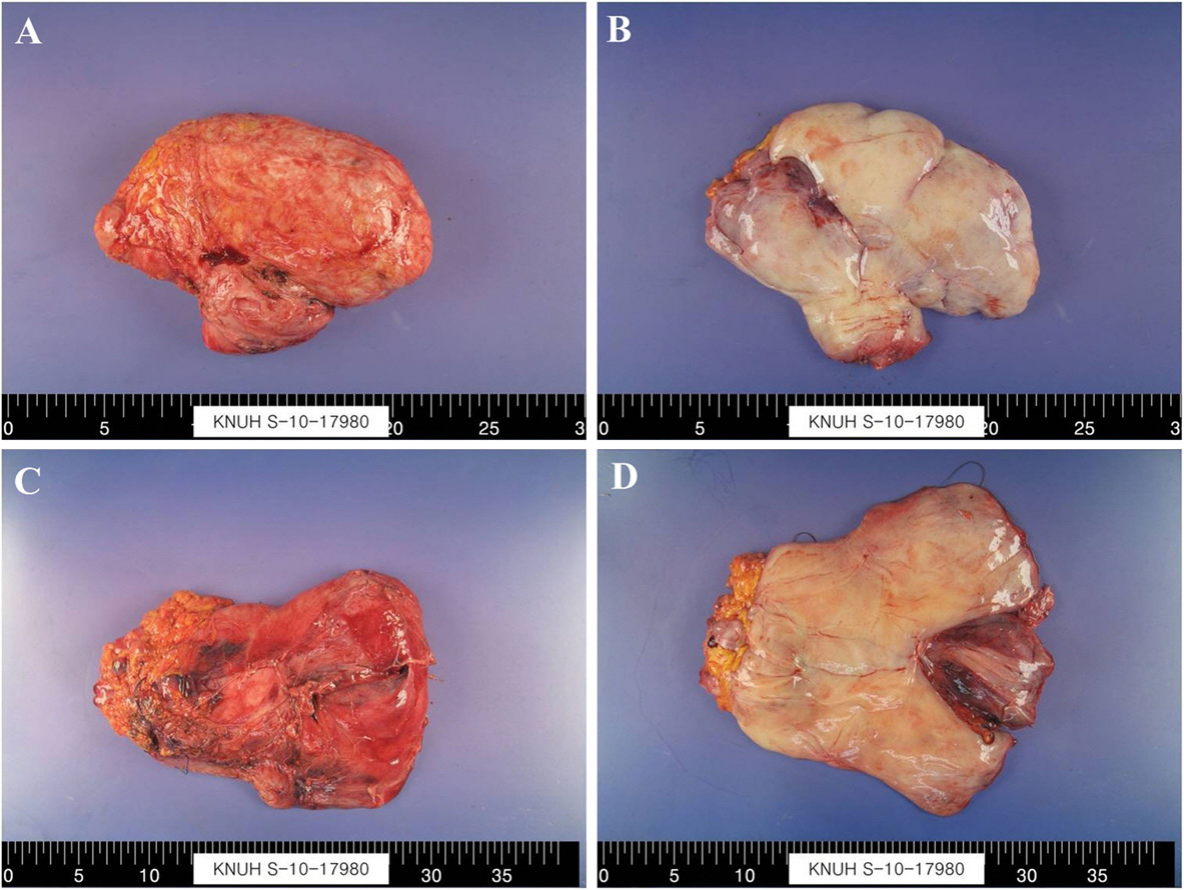
**Macroscopic findings.** (**A**) Upper retroperitoneal mass. (**B**) Section of retroperitoneal mass. (**C**) Lower perineal mass. (**D**) Section of perineal mass.

A rectal tube and urinary catheter were inserted under general anesthesia. The patient was placed in the prone position. An inverted Y-shaped skin incision centered on the lower portion of the sacrum was created to expose the posterior part of the sacrum and the coccyx (Figure 2A). The coccyx and lower sacrum were removed, revealing the retroperitoneal mass. The mass was well encapsulated. While palpating the rectal tube and urinary catheter, the surrounding organs and retroperitoneal mass were carefully dissected down to the stalk area where the perineal mass and retroperitoneal mass joined each other. To prevent tumor spread after excising the stalk, the cut plane of the stalk of the perineal mass was covered using a small laparoscopic endo-bag (Figure 3A, B). After inserting a drain, the cut plane of the buttock was sutured. With the patient in a dorsal lithotomy position, the perineal mass was simply excised and extracted (Figure 3C, D). Another drain was inserted in the perineum (Figure 2B).

Macroscopically, the tumor had a smooth surface, well-encapsulated upper portion, and partially encapsulated portion in the buttock region. The well-encapsulated retroperitoneal mass showed a glistening, gelatinous appearance and was homogenous in consistency without nodularity. However, the partially encapsulated subcutaneous lesion in the buttock area contained nodular and satellite-like lesions.

**Figure 4:**
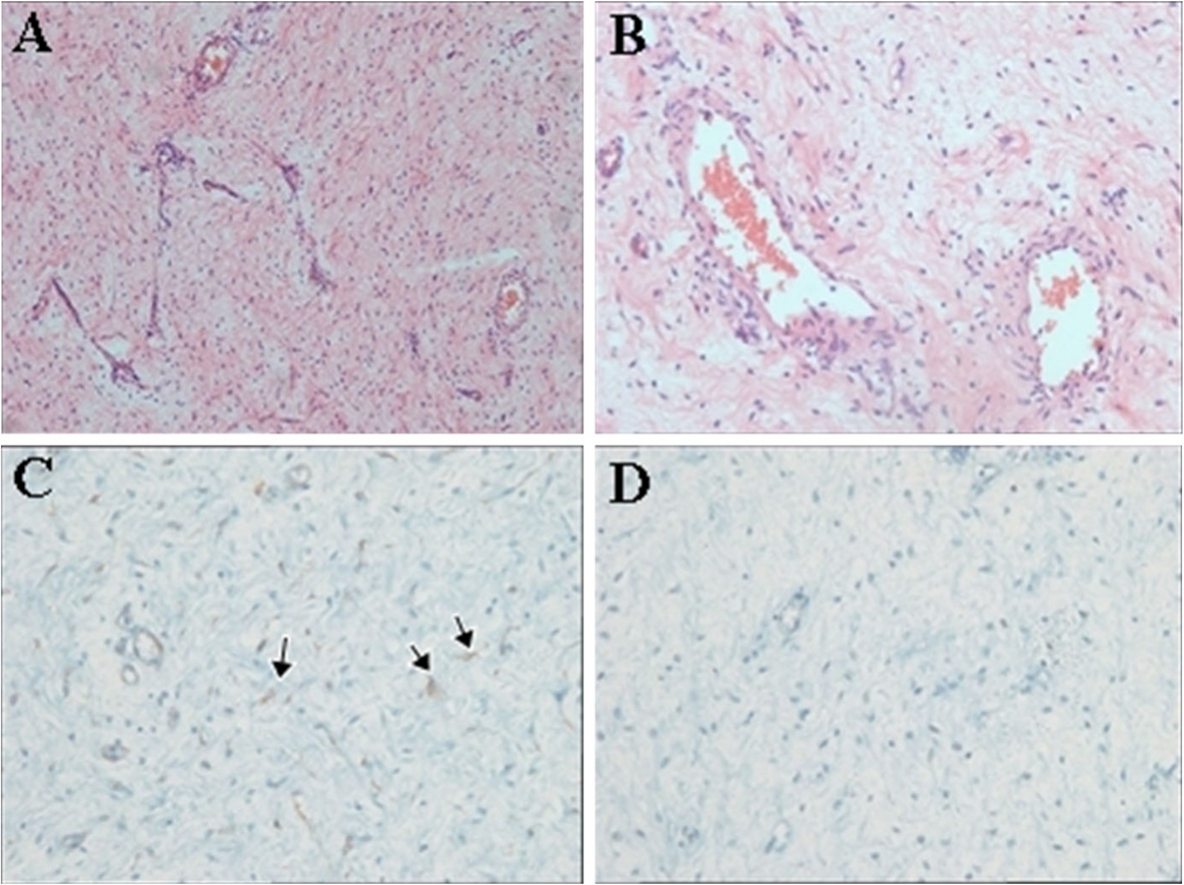
**Microscopic findings.** (**A**) The tumor shows satellite or spindle cells with a myxoid stroma and medium-sized prominent vessels (hematoxylin and eosin stain, ×100). (**B**) The tumor shows medium-sized vessels with perivascular hyalinization and thickened walls (hematoxylin and eosin stain, ×200). (**C**) A few tumor cells reveal positive reactivity for smooth muscle actin (SMA) immunohistochemical staining (arrows, SMA, ×400). (**D**) The satellite tumor cells reveal negative reactivity for S-100 protein immunohistochemical staining (S-100, ×400).

Microscopically, the tumor showed alternating hypercellular and hypocellular areas comprising small satellite to spindle cells with pale eosinophilic cytoplasm and a myxoid stroma with prominent vessels (Figure 4A). The tumor cells were cytologically bland, and mitosis was not present. Stromal vessels were medium-sized and thick-walled and showed perivascular hyalinization (Figure 4B). Immunohistochemical staining of the tumor cells revealed positive reactivity for smooth muscle actin (Figure 4C) but negative reactivity for S-100 protein (Figure 4D). Estrogen receptors and progesterone receptors were identified in the tumor tissue. Despite the poor circumscription of the mass, the resection margins were free from tumor extension with the exception of one close lateral margin of less than 1mm from the tumor extension. The drains were removed on the 10th postoperative day, and the patient was discharged on the 14th postoperative day. Three years after surgery, there was no recurrence.

## Discussion

Although AAA mostly occurs in women of reproductive age, it also occurs in men or children before puberty. It mainly occurs in urogenital organs such as the perineum, vagina, bladder, scrotum, and retroperitoneum, but may also occur in the larynx and orbit [4-6]. AAA is very difficult to diagnose before surgery. Radiological examinations such as ultrasonography, CT, and MRI are helpful in estimating the size of the tumor and degree of infiltration into the surrounding tissues and determining the range and method of surgery [7]. On CT, the tumor has well-defined margins and less attenuation than that of muscle. On T2-weighted MRI, the tumor has high signal intensity [8]. The best treatment of choice is surgical excision with tumor-free margins. However, the tumor recurs in approximately 70 percent of cases, even when the margin has been sufficiently excised [9]. Postoperative adjuvant therapy is also necessary, especially in cases of recurrent or residual tumors. It has been suggested that the growth of AAA is associated with stimulation by sex hormones, especially estrogen. Based on these reports, GnRH analogs have been used in estrogen- and progesterone-receptor-positive recurrent or residual tumors [10,11]. Radiotherapy has been also performed, but its effect is not clear [3].

There is no definite treatment modality with the exception of radical excision with tumor-free margins. The size of AAA varies from less than 5cm to 60cm, and large tumors frequently show retroperitoneal involvement. Cosmetic incisions are difficult to make in young women who undergo removal of bulky retroperitoneal masses via laparotomy. The patient in the present case was a 34-year-old woman, and she did not want to undergo laparotomy with a midline incision. If intra-abdominal organs are displaced and the peritoneum is dissected before the retroperitoneal mass removal, the risk of damage to adjacent organs and postoperative intraperitoneal adhesion will increase. In cases involving retroperitoneal masses close to the anus, the anal sphincter may be damaged; thus, local sphincter-preserving procedures are necessary. The trans-sacral approach to remove retroperitoneal masses was first proposed by Kraske in 1885 [12]. This method has since been used to remove colorectal tumors, pelvic bone tumors, and retroperitoneal soft tissue tumors. In 2003, Sonoda *et al*. [13] used the trans-sacral approach to remove a remnant cervix in a 71-year-old woman with endometrial cancer who had undergone previous laparotomy. He suggested that if radical resection is needed in an unusual situation, adaptation of different surgical approaches may be required. The herein-described inverted Y incision and trans-sacral approach can provide easy access to deep retroperitoneal AAA and reduce damage to neighboring organs.

## Conclusion

The inverted Y incision and trans-sacral approach performed by these authors is considered to result in minimal incision scars and provide easy access to tumors, avoiding damage to adjacent organs. These authors propose the above-mentioned procedure for bulky AAA involving the retroperitoneal space.

## Consent

Written informed consent was obtained from the patient for publication of this manuscript and any accompanying images. A copy of the written consent is available for review by the Editor-in-Chief of this journal.

## Competing interests

The authors declare that they have no competing interests.

## Authors’ contributions

DGH, GOC, and YSL performed the surgery. YLC and ISP reviewed the references and were major contributors in writing the manuscript. JYP performed the pathological diagnosis. All authors read and approved the final manuscript.
